# In-silico molecular design of heterocyclic benzimidazole scaffolds as prospective anticancer agents

**DOI:** 10.1186/s13065-019-0608-5

**Published:** 2019-07-11

**Authors:** Sumit Tahlan, Sanjiv Kumar, Kalavathy Ramasamy, Siong Meng Lim, Syed Adnan Ali Shah, Vasudevan Mani, Balasubramanian Narasimhan

**Affiliations:** 1 Faculty of Pharmaceutical Sciences, Maharshi Dayanand University, Rohtak, 124001 India; 2 Faculty of Pharmacy, Universiti Teknologi MARA (UiTM), 42300 Bandar Puncak Alam, Selangor Darul Ehsan Malaysia; 3 Collaborative Drug Discovery Research (CDDR) Group, Pharmaceutical Life Sciences Community of Research, Universiti Teknologi MARA (UiTM), 40450 Shah Alam, Selangor Darul Ehsan Malaysia; 4 Atta-ur-Rahman Institute for Natural Products Discovery (AuRIns), Universiti Teknologi MARA, Puncak Alam Campus, 42300 Bandar Puncak Alam, Selangor Darul Ehsan Malaysia; 5 Department of Pharmacology and Toxicology, College of Pharmacy, Qassim University, Buraidah, 51452 Kingdom of Saudi Arabia

**Keywords:** Benzimidazoles, Anticancer activity, Docking, CDK-8, ER-alpha, ADME

## Abstract

Benzimidazole is a valuable pharmacophore in the field of medicinal chemistry and exhibit wide spectrum of biological activity. Molecular docking technique is routinely used in modern drug discovery for understanding the drug-receptor interaction. The selected data set of synthesized benzimidazole compounds was evaluated for its in vitro anticancer activity against cancer cell lines (HCT116 and MCF7) by sulforhodamine B (SRB) assay. Further, molecular docking study of data set was carried out by Schrodinger-Maestro *v11.*5 using CDK-8 (PDB code: 5FGK) and ER-alpha (PDB code: 3ERT) as possible target for anticancer activity. Molecular docking results demonstrated that compounds **12**, **16**, **N9**, **W20** and **Z24** displayed good docking score with better interaction within crucial amino acids and corelate to their anticancer results. ADME results indicated that compounds **16**, **N9** and **W20** have significant results within the close agreement of the Lipinski’s rule of five and Qikprop rule within the range and these compounds may be taken as lead molecules for the discovery of new anticancer agents.
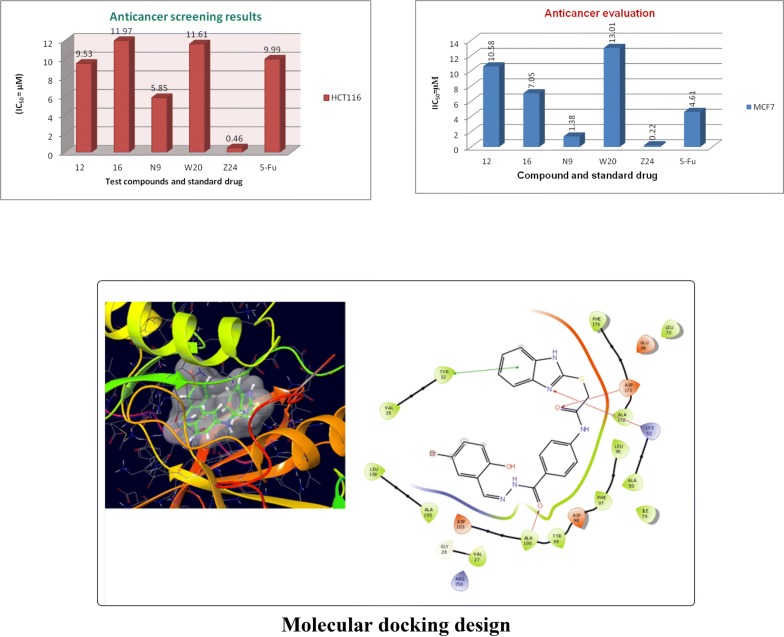

## Introduction

**Fig. 1:**
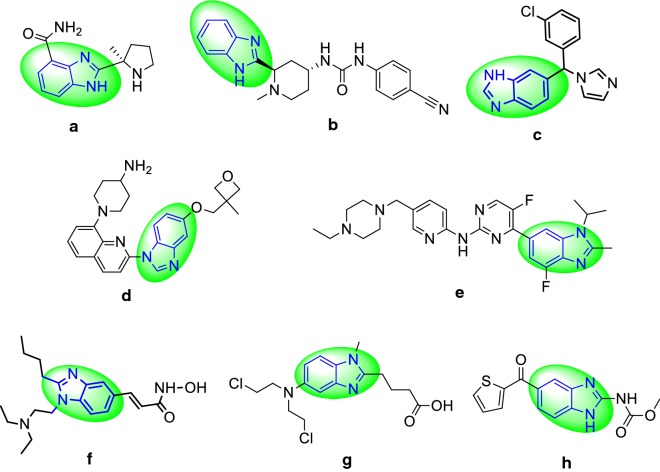
The selected marketed drugs having benzimidazole moiety

Benzimidazole is an important structural motif found in extensive number of natural and pharmacologically active compound [1]. The benzimidazole ring itself is an urgent pharmacophore in present day and has been used as privileged scaffolds to synthesize selective drugs of interest in medicinal field including antiulcer [2], antioxidant [3], HIV-RT inhibitor [4], anticancer [5], antihelmintic [6], antimicrobial [7], antihistamine [8] etc. The selected marketed drugs having benzimidazole moiety (Fig. 1) i.e. veliparib (a), glasdegib (b), liarozole (c), crenolanib (d), abemaciclib (e), pracinostat (f), bendamustine (g) nocodazole (h).

Presently used chemotherapeutic agents restrain the growth of tumour through suppression of DNA replication and transcription. Nevertheless, the attempt of discovering new curative anticancer agents in last decade has led to targets of specific molecular modifications in tumour cells. The new approach now focuses mainly on the development of small biologically active molecules containing significant activity without toxicity related to the usual chemotherapy [9]. The human CDK8 protein which is a part of the RNA polymerase has been one of the proteins responsible for acute lymphoblastic leukaemias.

CDK activity is controlled by association with regulatory subunits (cyclins) and CDK inhibitor proteins, by their phosphorylation state and by ubiquitin-mediated proteolysis. Since the loss of cell cycle control leading to deregulated cell proliferation is one of the hallmarks of cancer, it is anticipated that the inhibition of CDKs will provide an effective approach to control tumor growth and therefore have an impact on cancer therapy. Inhibition of CDKs has been studied by many organizations and has been achieved using a variety of structural templates with varying degrees of selectivity and activities [10].

Western blotting analysis was used to demonstrate that three hits target CDK8 in HCT 116 colorectal cancer cells. The results showcase the successful application of virtual screening cascades to identify CDK8-targeted scaffolds that can be developed into a drug discovery program. CDK8 is a cyclin-dependent kinase that forms part of the Mediator complex, which itself regulates the transcriptional activity of RNA polymerase II. A number of studies have shown that CDK8 modulates the transcriptional output from distinct transcription factors involved in oncogenic control. These factors include the Wnt/β-catenin pathway, Notch, p53 and TGF-*β*. CDK8 has been found to be amplified and over expressed in colon cancer. In this context, CDK8 has been reported to act as a colon cancer oncogene. The role of CDK8 in both cellular signaling and colon cancer have relied upon RNAi mediated suppression of CDK8 and on the use of a kinase dead mutant CDK8. In order to more fully investigate the role of CDK8 in colon cancer, we aimed to develop a potent and selective small molecule inhibitor of CDK8 [11].

CDK-8 is a heterodimeric kinase protein responsible for regulation of cell cycle progression, transcription and other functions. CDKs require cyclin that provides additional sequences for enzymatic potential. All the CDKs (1, 4, 5, 7, 8, 9 and 11) have a two-lobed structure-*N*-terminal having beta sheets and C-terminal composed of α-helices [12, 13].

Estrogen signaling is essential in the initiation and development of human breast cancer. In the past several decades, extensive efforts have been dedicated to understand the underlying mechanisms of this important signaling pathway in human breast cancer, which have facilitated the development of anti-estrogen therapy, the first targeted therapy for human cancer. Estrogen biology is exceedingly complex and important in the development and function of numerous tissues and physiological phenomena [14, 15]. Computational approaches i.e. molecular docking used for modern drug discovery design for the medicinal drug [16]. Drug molecules might fail during development because of several reasons but as found by the researchers one of the major reasons of failures is related with poor pharmacokinetic: ADME properties [17]. Drug toxicity is the one of the major factors to withdraw drug from the market. Therefore, ADME properties are the crucial determinants for the clinical success of the drug [18]. Now these days, computer based drug design are employed to determine the ADME profile of the compound. ADME modeling has attracted the considerable attention of the pharmaceutical researchers for the drug discovery as they are high-throughput in nature and cost effective [19].

Recently, a greater emphasis has been given towards the researches on complementary and alternative medicine that deals with cancer management. The present study helps us to understand the interaction between the benzimidazoles and receptors

(CDK-8 and ER-alpha) and also explore their binding mode by in silico molecular design.

## Experimental

### Materials and methods

#### Data set

The data set of selected benzimidazole compounds have exhibited better anticancer activity towards human colorectal carcinoma cancer cell line (HCT116) was selected from the earlier study reported by Tahlan et al. [5, 7, 9, 20, 21]. The molecular structures of all reported compounds were drawn in ChemDraw Ultra software 12.0. The data set of selected benzimidazole compounds with their anticancer activity results is shown in Table 1 (S. No. 1 to 18).

**Table 1:**
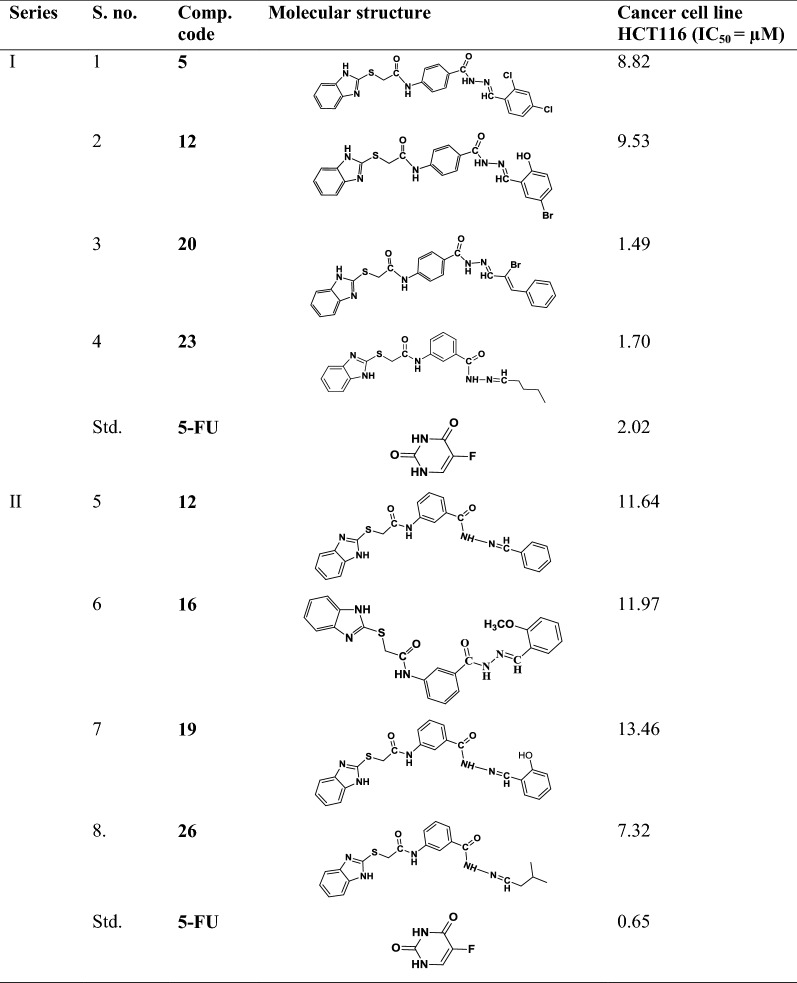

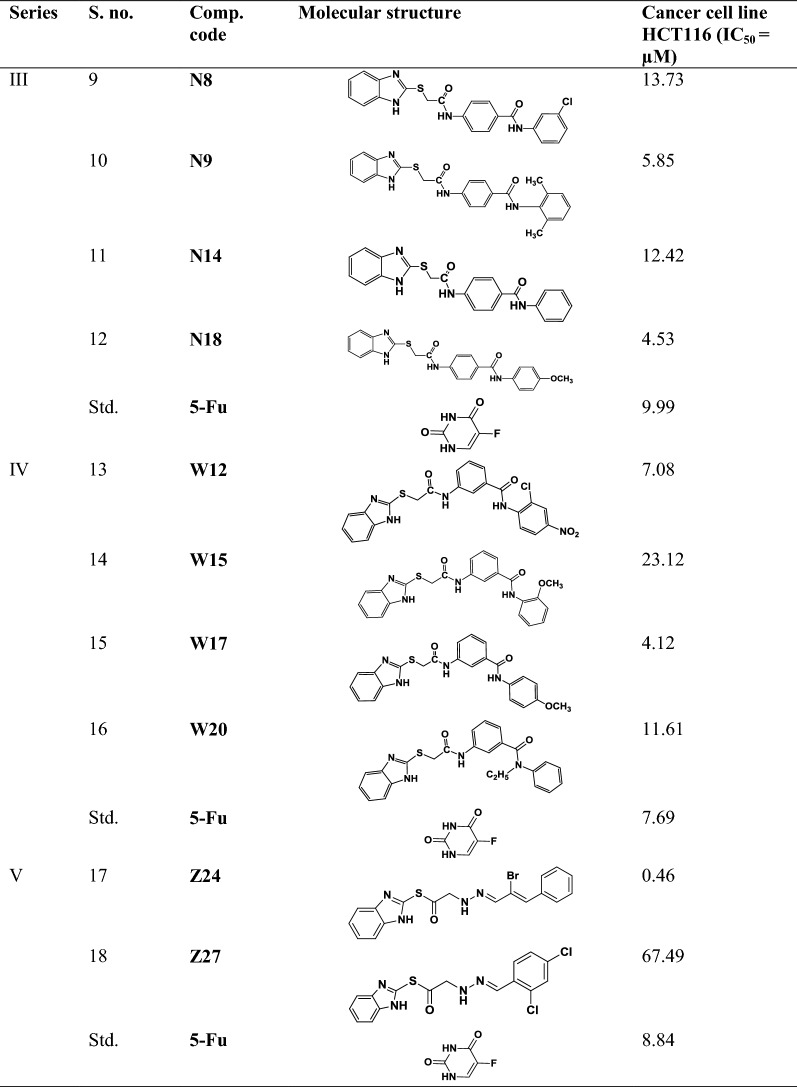
Data set of selected benzimidazole compounds with their anticancer results

#### Physicochemical and spectral interpretation data of most active compounds

##### 2-((1*H*-Benzo[d]imidazol-2-yl)thio)-*N*-(4-(2-(5-bromo-2-hydroxybenzylidene)hydrazine-carbonyl)phenyl)acetamide (**12**)

M.pt. °C: > 300; R*f* value: 0.52; % yield: 85.87; IR: 3077 (C–H str.), 1439 (C=C str.), 1669 (–CONH str.), 1362 (N=CH str.), 1317 (C–N str.), 730 (C–S str.), 3629 (O–H str.), 625 (C–Br str.); ^1^H-NMR: 3.84 (s, 2H, CH_2_), 4.30 (s, 1H, OH), 4.31 (s, 1H, NH of imidazole), 6.77–7.93 (m, 11H, Ar–H), 7.92 (s, 2H, (CONH)_2_), 7.93 (s, 1H, N=CH); ^13^C-NMR: 36.76, 111.0, 118.3, 118.8, 119.4, 121.0, 122.8, 125.8, 130.0, 132.1, 135.9, 139.1, 143.2, 150.1, 161.2, 158.1, 167.2; Anal. Calcd. for C_23_H_18_N_5_O_3_SBr: C, 52.68; H, 3.46; N, 13.36; Found: C, 52.72; H, 3.50; N, 13.40; MS ES + (ToF): *m/z* 527 [M^+^+1].

##### 2-((1*H*-Benzo[d]imidazol-2-yl)thio)-*N*-(3-(2-(2-methoxybenzylidene)hydrazinecarbonyl)-phenyl)acetamide (**16**)

M.pt. °C: 122–125; R*f* value: 0.80; % yield: 75.42; IR: 3108 (C–H str.), 1606 (C=C str.), 1669 (–CONH str.), 1320 (N=CH str.), 1289 (C–N str.), 665 (C–S str.), 1247 (C–O–C str.), 2835 (C–H str., O–CH_3_); ^1^H-NMR: 7.04–8.26 (m, 12H, Ar–H), 4.31 (s, 1H, NH of imidazole), 3.85 (s, 2H, CH_2_), 7.99 (s, 2H, (–CONH)_2_), 8.26 (s, 1H, N=CH), 3.85 (s, 3H, CH_3_); ^13^C-NMR: 36.6, 56.2, 112.5, 117.7, 120.3, 121.1, 122.2, 123.6, 124.7, 129.5, 129.7, 133.4, 139.5, 150.1, 159.2, 165.9, 167.5; Anal. Calcd. for C_24_H_21_N_5_O_3_S: C, 62.73; H, 4.61; N, 15.24; Found: C, 62.77; H, 4.65; N, 15.28; MS ES + (ToF): *m/z* 462 [M^+^+1].

##### 4-(2-(1*H*-Benzo[d]imidazol-2-ylthio)acetamido)-*N*-(2,6-dimethylphenyl)benzamide (**N9**)

M.pt. °C: 207–210; R*f* value: 0.56; % yield: 64.03; IR: 3018 (C–H str.), 1598 (C=C str.), 1668 (–CONH str.), 1360 (N=CH str.), 1281 (C–N str.), 713 (C–S str.), 2915 (C–H str., –CH_2_–), 2948 (C–H str., CH_3_); ^1^H-NMR: 7.16–7.96 (m, 11H, Ar–H), 4.36 (s, 1H, NH of imidazole), 7.97 (s, 2H, (CONH)_2_), 2.53 (s, 6H, (–CH_3_)_2_); ^13^C-NMR: 39.0, 118.3, 121.6, 125.4, 130.4, 142.8, 149.7, 166.6, 166.8; Anal. Calcd. for C_24_H_22_N_4_O_2_S: C, 66.96; H, 5.15; N, 13.01; Found: C, 66.99; H, 5.19; N, 13.05; MS ES + (ToF): *m/z* 431 [M^+^+1].

##### 3-(2-(1*H*-Benzo[d]imidazol-2-ylthio)acetamido)-*N*-ethyl-*N*-phenyl-benzamide (**W20**)

M.pt. °C: 216–219; R*f* value: 0.42; % yield: 83.22; IR: 3096 (C–H str.), 1598 (C=C str.), 1336 (N=CH str.), 1304 (C–N str.), 1664 (–CONH str.), 701 (C–S str.), 2932 (C–H str., CH_3_), 2826 (C–H str., N–CH_3_), 2915 (C–H str., –CH_2_–); ^1^H-NMR: 7.12–8.33 (m, 13H, Ar–H), 4.31 (s, 1H, NH of imidazole), 7.82 (s, 2H, (CONH)_2_), 2.51 (q, 2H, CH_2_); ^13^C-NMR: 36.1, 119.7, 119.7, 121.4, 123.1, 124.2, 129.0, 131.3, 139.0, 149.7, 166.4, 167.0; Mol. Formula C_24_H_22_N_4_O_2_S; Elem. Anal. Calcd. C, 66.96; H, 5.15; N, 13.01; Found C, 66.93; H, 5.19; N, 13.04; MS: *m/z* 431 [M^+^+1].

##### 1*H*-Benzo[d]imidazol-2-yl2-(2-(2-bromo-3-phenylallylidene)hydrazinyl)ethanethioate (**Z24**)

M.pt. °C: 160–163; R*f* value: 0.51; % yield: 78.68; IR: 3087 (C–H str.), 1601 (C=C str.), 1713 (–CO str.), 1354 (C=N str.), 1337 (C–N str.), 1176 (N–N str., hydrazide), 694 (C–S str.), 2843 (C–H str., –CH_2_–), 684 (C–Br str.), 1623 (conjugated C=C and phenyl subst. C=C); ^1^H-NMR: 7.13–7.94 (m, 9H, Ar–H), 7.06 (s, 1H, NH of imidazole), 2.00 (s, 2H, CH_2_), 7.06 (s, 1H, NH), 12.55 (s, 1H, N=CH), 7.19 (s, 1H, Br–C=CH); ^13^C-NMR: 109.4, 122.1, 123.8, 128.6, 128.9, 130.5, 131.3, 132.2, 132.8, 150.1, 187.8; Mol. Formula C_18_H_15_N_4_OSBr; Elem. Anal. Calcd. C, 52.06; H, 3.64; N, 13.49; Found C, 52.02; H, 3.68; N, 13.45; MS: *m/z* 416 [M^+^+1].

### Molecular docking

#### Preparation of ligand structure

The ligand structures of the data set were prepared by LigPrep module of Schrodinger *v11.5*. To give the best results, the structures that are docked must be good representations of the actual ligand structures as they would appear in a protein–ligand complex. This means that for Glide docking the structure must meet the following conditions. They must be three-dimensional (3D) form. Glide only modifies the torsional internal coordinates of the ligand during docking, so the rest of the geometric parameters must be optimized beforehand. They must each consist of a single molecule that has no covalent bonds to the receptor, with no accompanying fragments, such as counter ions and solvent molecules. They must have all their hydrogen (filled valences). They must have an appropriate protonation state for physiological pH values (around 7) [22, 23].

#### Preparation of protein structure

**Fig. 2:**
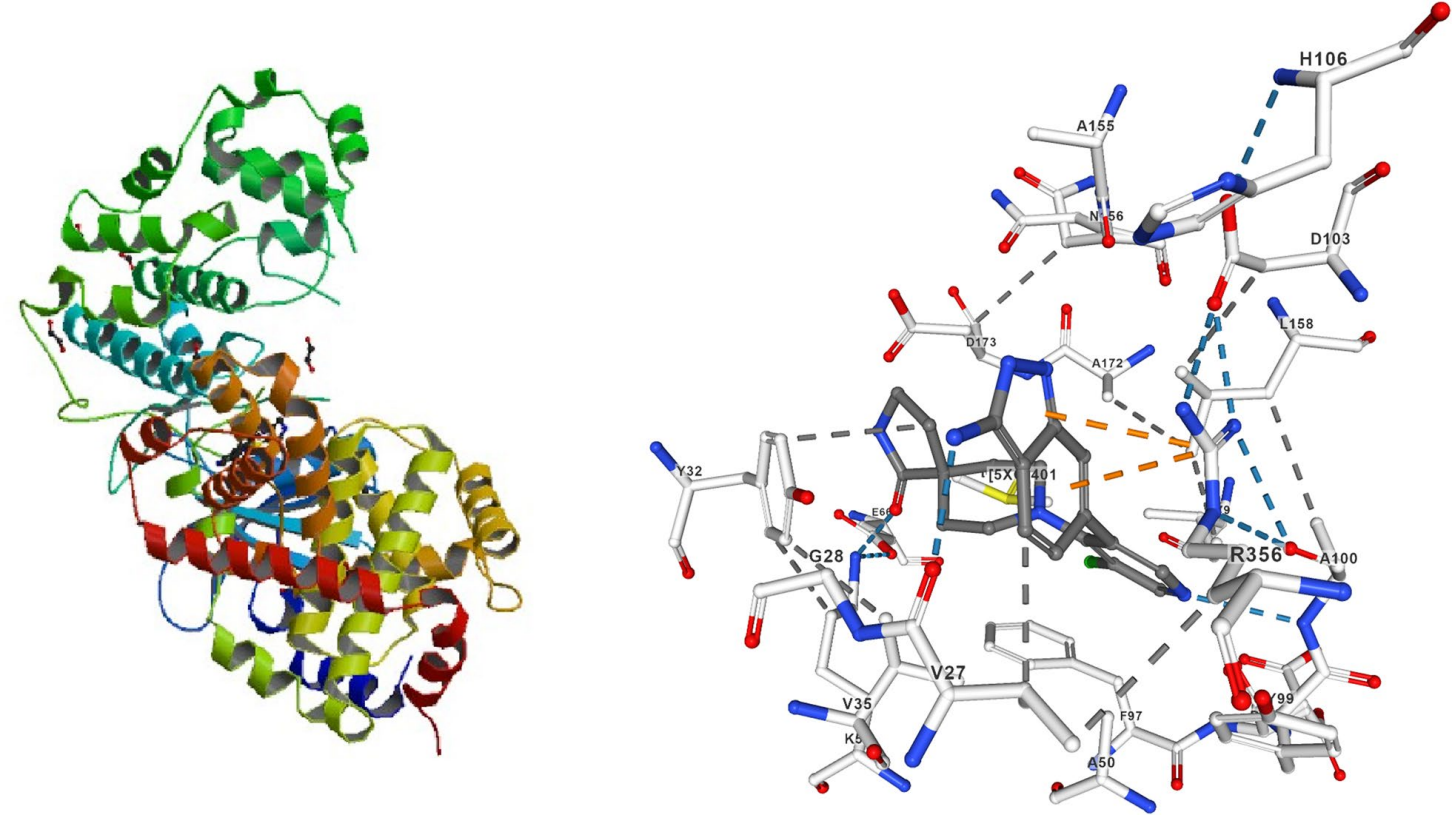
Protein structure with 5XG ligand (5FGK)

**Fig. 3:**
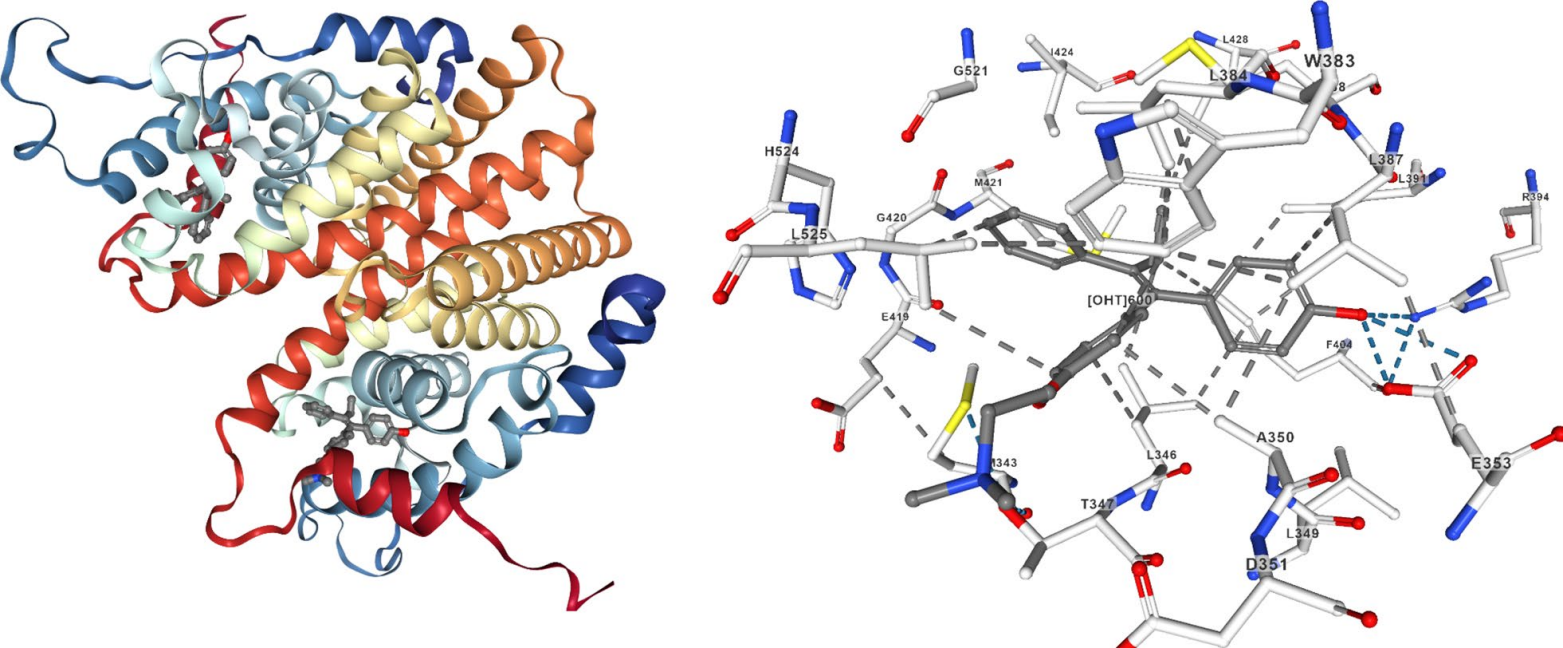
Protein structure with OHT ligand (3ERT)

The selected proteins i.e. human cyclin-dependent kinase CDK-8 (PDB code: 5-FGK) and ER-alpha (PDB code: 3ERT) (Figs. 2 and 3) were obtained from the protein data bank (PDB) (http://www.rcsb.org/pdb/home/home.do). The imported typical structure file of protein from the protein data bank is not suitable for immediate use to carry out the molecular docking study. A typical PDB structure file consists of heavy atoms and may include a co-crystallized ligand, water molecules, metal ions and cofactors. The ligand and ligand-receptor complex is suitable for use with other Schrödinger modules. The protein structure was prepared using the protein preparation wizard (preprocessed, optimized and minimized) in the Schrodinger software graphical user interface *Maestro v11.5* [24].

#### Preparation of grid

Grid generation is done using receptor grid generation module of maestro version *11.5.* A grid is generated around the binding site already occupied by the co-crystallized ligand so that co-crystallized ligand can be excluded and new compounds can be attached to the same binding site [25].

#### Docking

Molecular docking study was applied to investigate the binding mode of compound with selected PDB ID for cancer cell lines. Docking score obtained from GLIDE (maestro *v11.5*) and binding site was targeted and the grid was created. The active site grid covered the important amino acids interacting with receptor. The 3D structure of the protein was obtained from protein data bank using their specific (PDB code: 5FGK and ER-alpha PDB code: 3ERT). A data set of benzimidazoles was used as ligands and their structures were drawn using the workspace of Maestro and were converted to 3D form for the docking studies. The collected ligands were prepared for docking. Then the prepared ligands were docked into the generated grid in the prepared protein. The best docked pose with lowest glide score value was recorded for each ligand. Extra precision (XP) was performed using Schrödinger-*maestro v11.5 (2018*-*1)* [26–28].

#### ADME prediction

Theoretical calculation of the ADME properties of data set was calculated by QikProp. Nearly eleven physically significant descriptors and pharmacologically properties of the compounds were analyzed by QikProp. Aqueous solubility of compounds plays a key impact on many ADME associated properties like uptake, distribution, transport and ultimately bioavailability. The benzimidazole derivatives solubility values were within the range. Finally, the Lipinski’s rule of five and Qikprop rule within the range for the benzimidazole derivatives and thus making these derivatives as suitable drug candidates [12].

#### Anticancer evaluation (IC_50_)

The anticancer activity was determined by Sulforhodamine-B (SRB) assay. Briefly, HCT116 and MCF7 cancer cell lines were seeded onto the 96 well plate at 2500 cells/well. The cells were allowed to attach overnight before being exposed to the respective compounds (0.01–100 µg/mL) for 72 h. The highest concentration of each compound tested (100 µg/mL) contained only 0.1% DMSO (non-cytotoxic) SRB assay was then performed whereby the cells were fixed using trichloroacetic acid for 30 min at 4 °C and stained with 0.4% (w/v) SRB mixed with 1% acetic acid for 15 min. After five washes with 1% acetic acid solution, the protein-bound dye was extracted with 10 mM tris base solution. Optical density was read at 570 nm and IC_50_ of each compound was determined. Anticancer results were presented as mean IC_50_ of at least triplicates (Tables 3 and 4) [29].

## Results and discussion

### Target identification

Kinase inhibitors are very efficacious for the treatment of cancer especially targeting specific mutations that chiefly drive tumorigenesis. They are categorized according to their capacity to catalyze the transfer of the terminal phosphate of ATP to the substrates that usually contain a serine, threonine or tyrosine residue [30]. Cyclin-dependent kinases (CDKs) are a family of key regulatory proteins that oversee diverse cellular events and their main involvement is in the cell cycle and transcription. Given the fundamental biological roles CDKs perform, it is not surprising that their aberrant activities are a common feature of many diseases, especially cancer. CDKs are a family of serinethreonine protein kinases that govern the initiation, progression and completion of the cell cycle. Activity of the CDKs allows the orderly transition between phases of the cell cycle. Inhibition of cell cycle progression and apoptosis are the most common causes of cell growth inhibition. Cell cycle progression is induced by various cell cycle proteins such as CDKs and cyclins as they are the key regulators of cell cycle [31]. Existing CDK8 X-ray crystal structures have unresolved regions in the vicinity of the ATP-binding site. Thus, homology modeling was used to generate two complete and optimal structural models. The target structure should be determined experimentally by either X-ray crystallography or nuclear magnetic resonance, which can be downloaded from PDB; however, docking has been performed successfully in comparison to homology models or threading. The model should have good quality. It can be tested using validation software such as Molprobity [32].

#### Molecular docking results

**Fig. 4:**
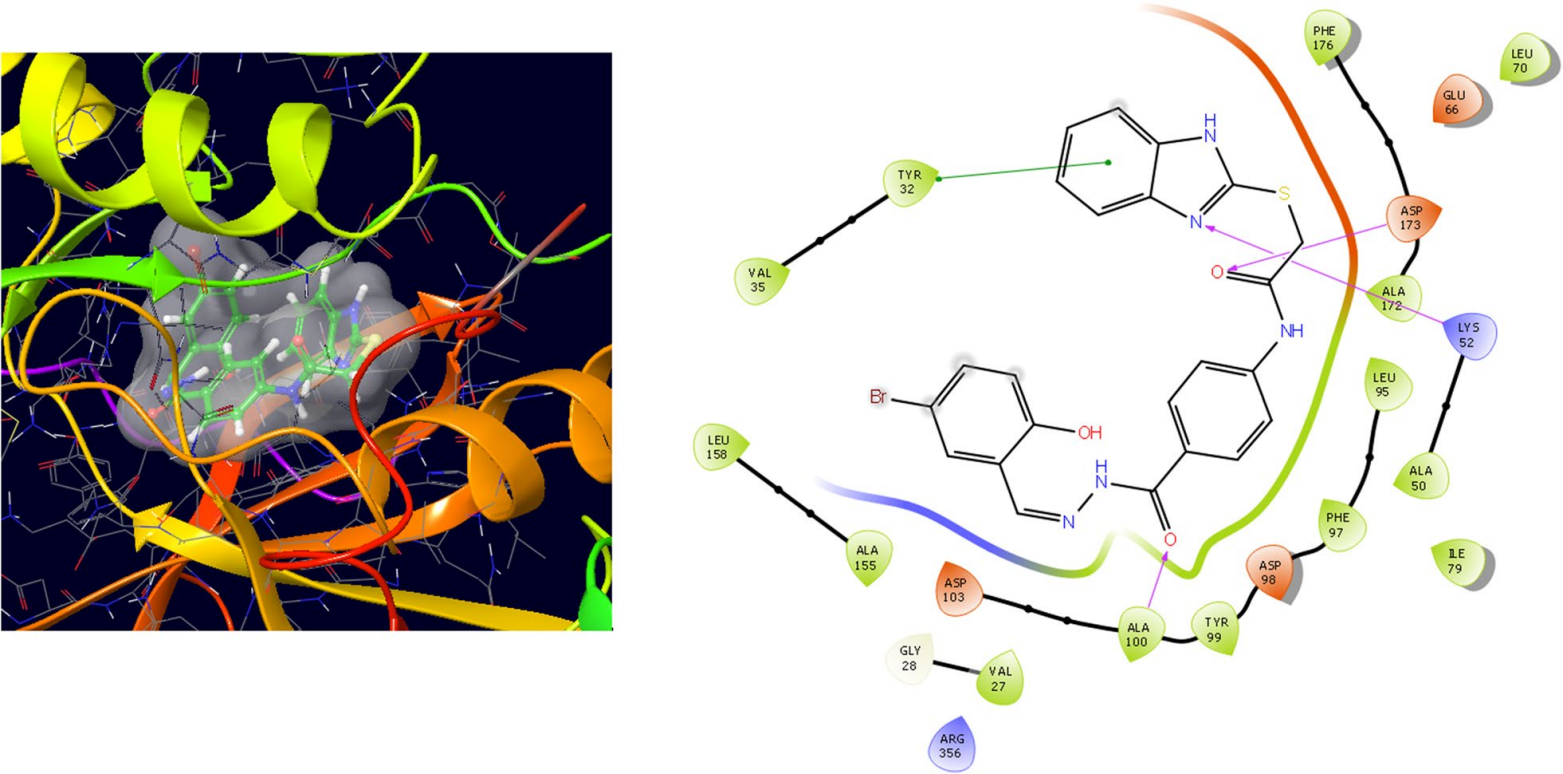
Binding surface and ligand interaction diagram of compound **12**

**Fig. 5:**
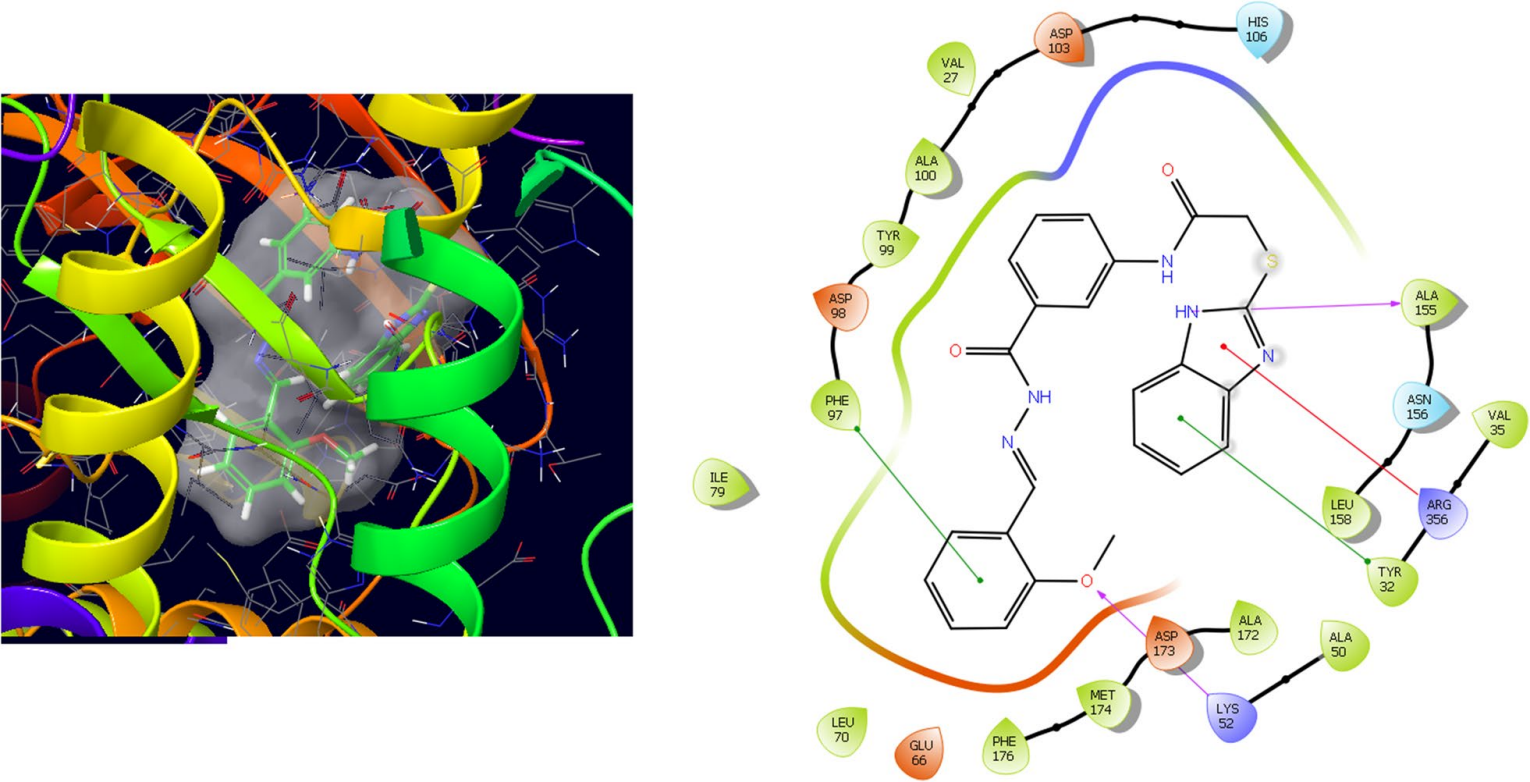
Binding surface and ligand interaction diagram of compound **16**

**Fig. 6:**
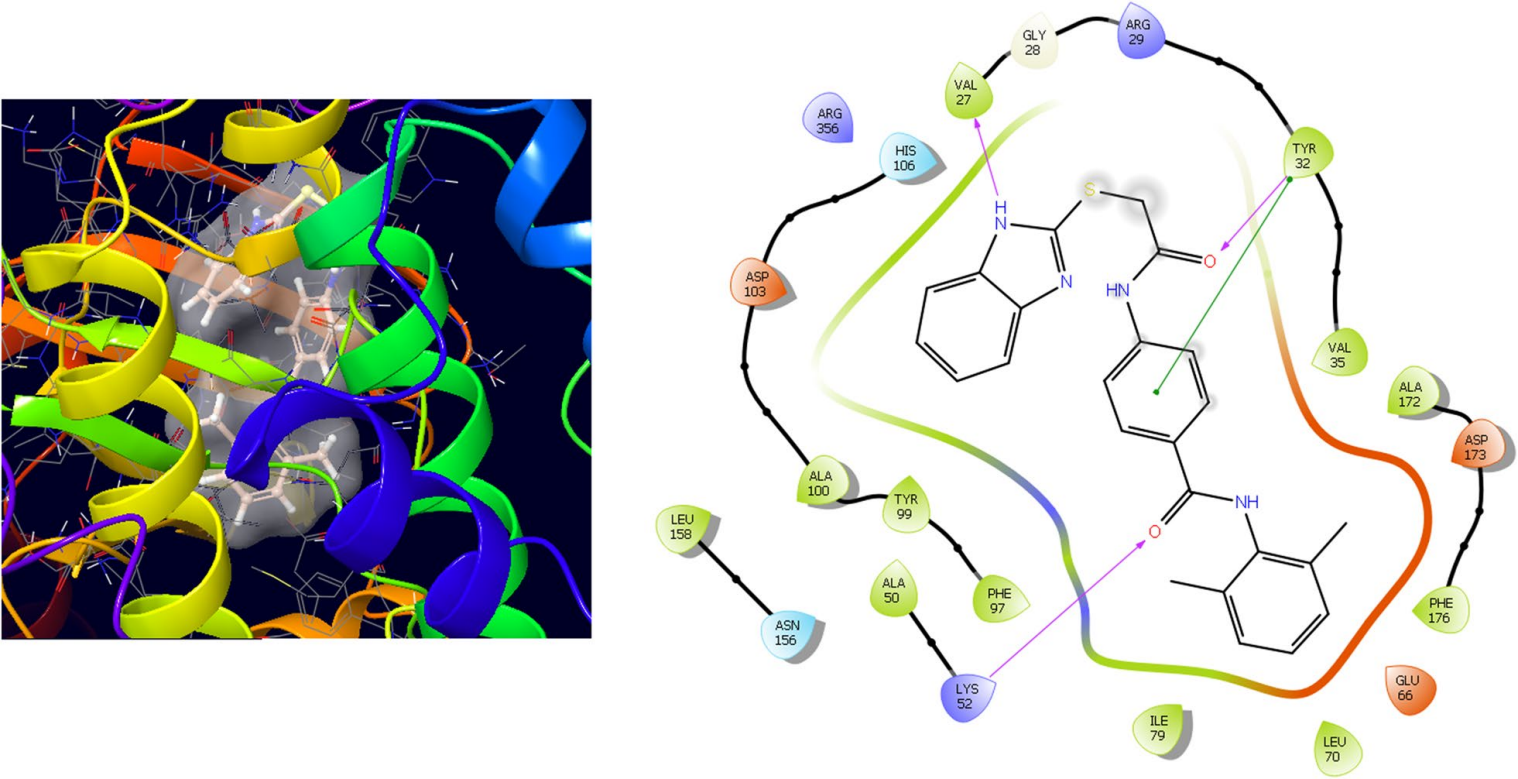
Binding surface and ligand interaction diagram of compound **N9**

**Fig. 7:**
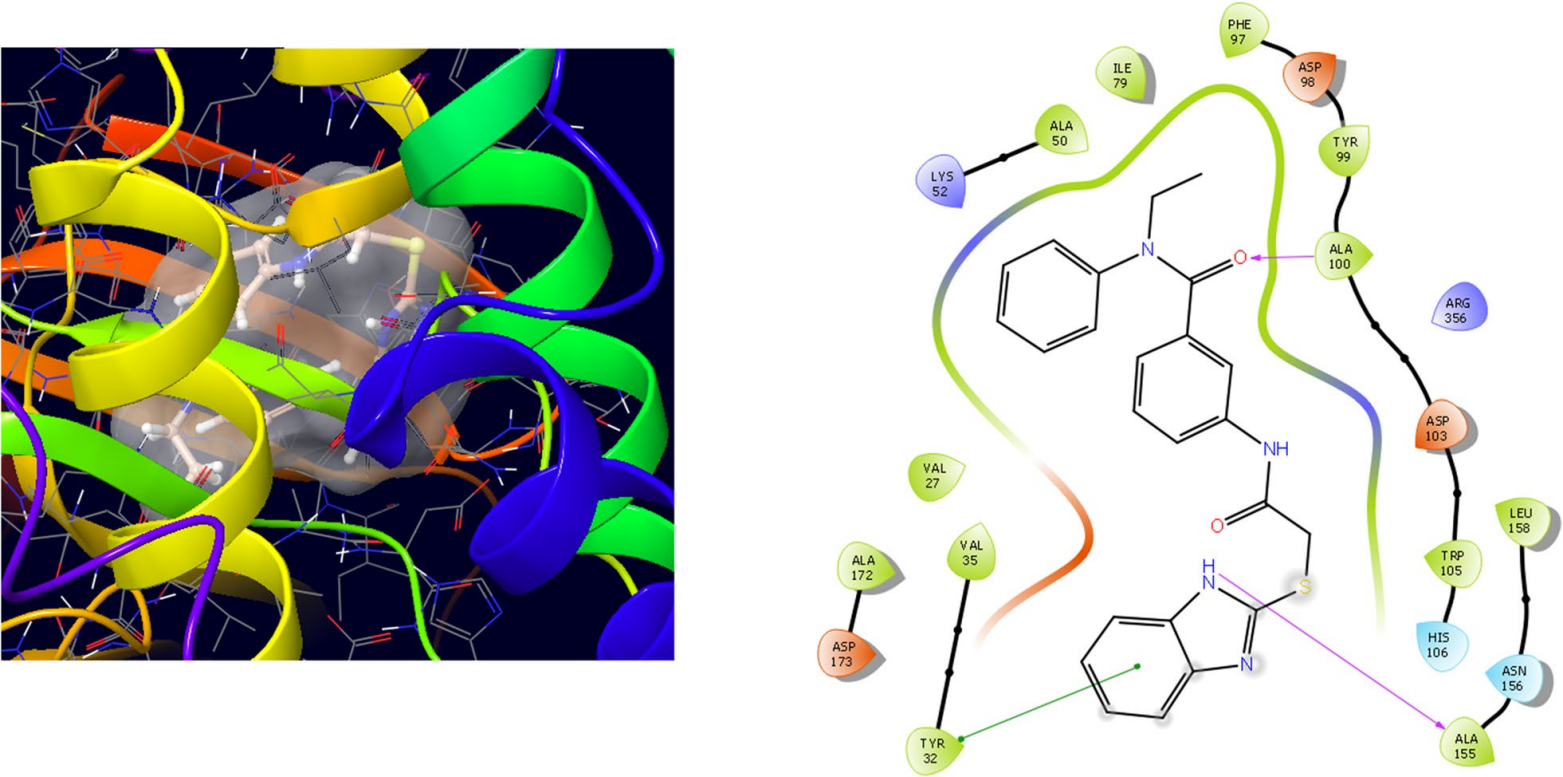
Binding surface and ligand interaction diagram of compound **W20**

**Fig. 8:**
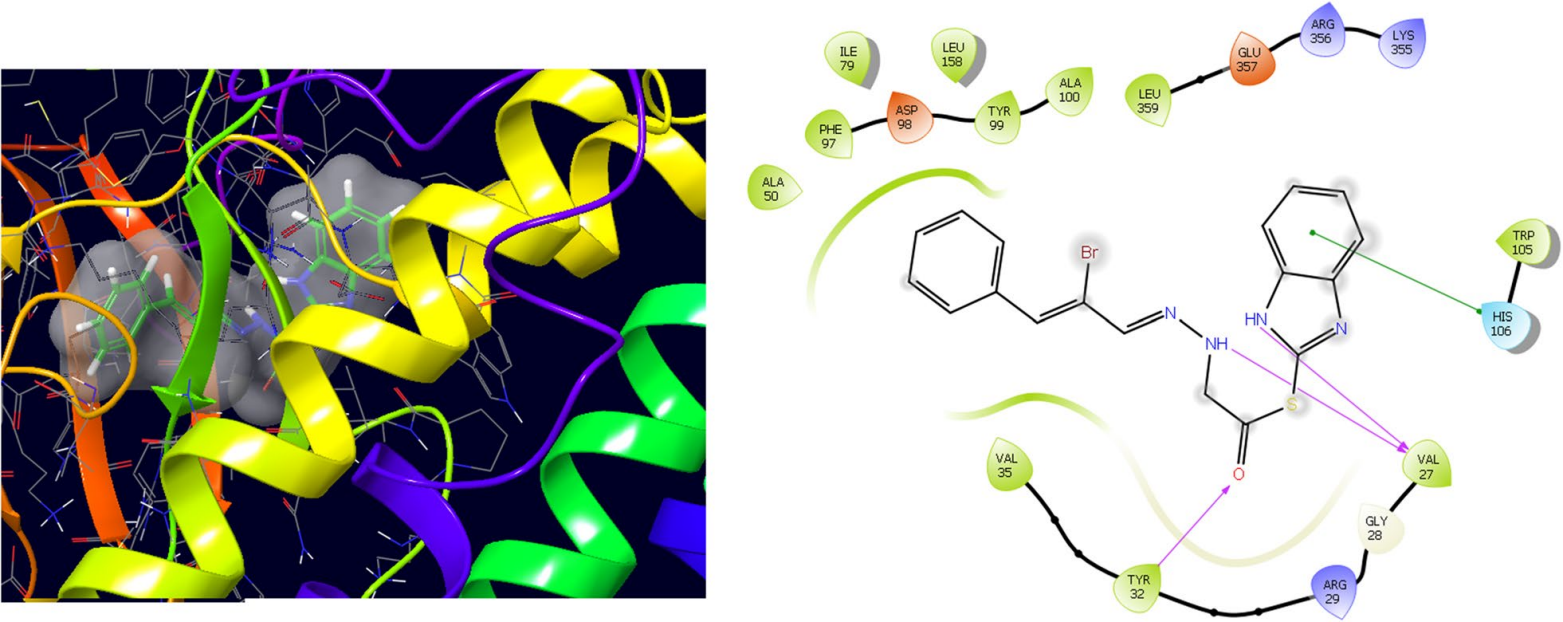
Binding surface and ligand interaction diagram of compound **Z24**

**Fig. 9:**
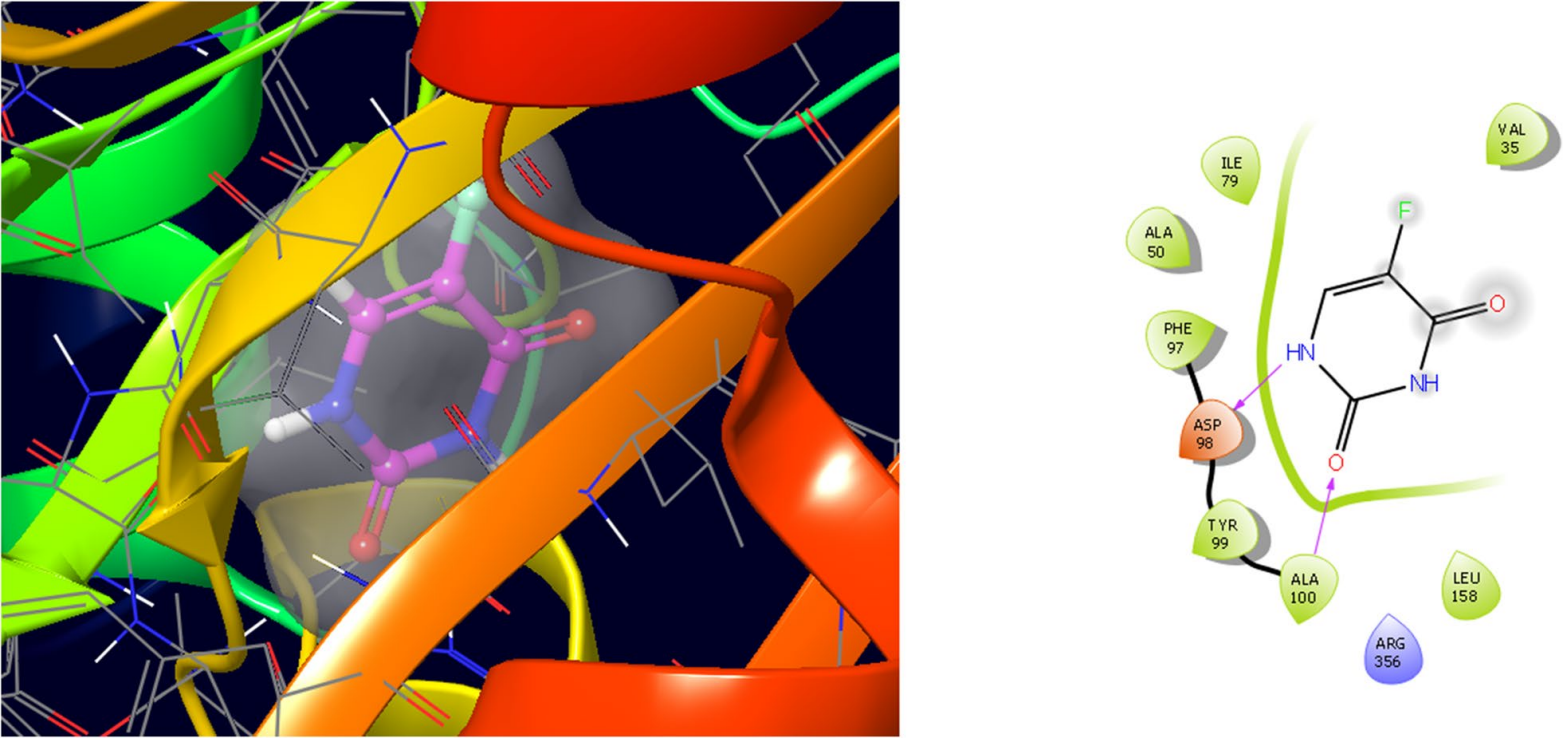
Binding surface and ligand interaction diagram of 5-fluorouracil

Molecular docking study was carried out to analyses the binding mode of the compounds against human colorectal carcinoma and breast adenocarcinoma cancer cell lines. Ligand interaction showed the binding mode of compound and standard drugs in the active site of CDK8 (PDB id: 5FGK) have good resolution about 2.36 Å, co-crystallized ligand (5XG) was selected for docking study. Root-mean-square deviation (RMSD) value of docked poses of native co-crystallized ligand is 0.10 Å, R-value free is 0.237 and ER-alpha (PDB id: 3ERT) have good resolution about 1.9 Å, co-crystallized ligand (OHT) was selected for docking study. RMSD value of docked poses of native co-crystallized ligand is 2.0 Å. R-value free is 0.262. Docking study of the data set showed good docking score and interaction with crucial amino acids residues in the binding pocket of the receptor (Table 2). The molecular docking results demonstrated in terms of negative energy value that the lower the binding energy value, best would be the binding affinity with the receptor [33]. Docking results with cdk-8 protein, compounds **12**, **16**, **N9**, **W20** and **Z24** were found to be best molecules and showed better docking score at target site of protein and displayed good to moderate anticancer activity against cancer cell line (HCT116). If we look into the binding mode of compound **12**, exhibited good docked score (− 8.907) and formation of hydrogen bond with amino acids i.e. Asp173, Ala100 and Lys52 with oxygen and nitrogen atoms. The binding mode of compound **16** have docking score (− 7.69) and developed of hydrogen bond with amino acids (Lys52 and Ala155) with oxygen atom of OCH_3_ and nitrogen atom of benzimidazole ring, respectively. The binding mode of compound **N9** exhibited good docking score (− 7.425) and prepared hydrogen bond with amino acids (Lys52, Tyr32 and Val27) with oxygen atom and nitrogen atoms of benzimidazole ring, respectively. Compound **W20** showed good docking score (− 9.686) and prepared hydrogen bond with amino acids (Ala100 and Ala155) with oxygen and nitrogen atoms of benzimidazole ring, respectively. Compound **Z24** displayed the good docking score (− 7.295) and developed hydrogen bond with amino acids (Tyr32 and Val27) with oxygen and nitrogen atoms, respectively. Standard drugs (5-fluorouracil) have good docking score (− 5.79) and formation of hydrogen bond with amino acids residues (Ala100 and Asp98) with oxygen and nitrogen atoms of 5-fluorouracil, respectively. These compounds showed better docked score than the standard drug and the docking results also correlate to their anticancer activity results. The docking results with interacting residues of the docked compounds and standard drug are shown in Table 3; binding surface (3D) and ligand interaction (2D) images are shown in Figs. 4, 5, 6, 7, 8, 9.

**Table 2:** Docking results of the selected benzimidazole compounds

Series	S. no.	Comp. code	CDK8 (HCT116)		3ERT (MCF7)	
			Docking score	Glide energy (kcal/mol)	Docking score	Glide energy (kcal/mol)
I	1	**5**	− 8.485	− 57.409	− 8.409	− 61.051
	2	**12**	− 8.907	− 57.165	− 8.825	− 63.027
	3	**20**	− 6.657	− 62.959	− 8.69	− 54.556
	4	**23**	− 6.795	− 52.624	− 8.301	− 57.817
II	5	**12**	− 6.627	− 52.907	− 8.361	− 54.128
	6	**16**	− 7.69	− 57.228	− 8.986	− 54.764
	7	**19**	− 6.778	− 64.087	− 8.365	− 56.448
	8	**26**	− 5.426	− 54.725	− 5.982	− 49.251
III	9	**N8**	− 6.148	− 56.817	− 7.878	− 58.473
	10	**N9**	− 7.425	− 53.041	− 6.748	− 49.725
	11	**N14**	− 5.787	− 47.184	− 7.983	− 50.79
	12	**N18**	− 5.37	− 57.631	− 7.665	− 55.517
IV	13	**W12**	− 7.736	− 58.249	− 7.781	− 54.771
	14	**W15**	− 5.558	− 52.555	− 7.884	− 51.905
	15	**W17**	− 7.554	− 54.696	− 8.642	− 52.047
	16	**W20**	− 9.686	− 52.697	− 7.703	− 58.783
V	17	**Z24**	− 7.295	− 47.998	− 7.275	− 45.298
	18	**Z27**	− 5.616	− 41.802	− 7.98	− 42.365
	Std.	**5-Fu**	− 5.79	− 21.629	− 3.414	− 24.58

**Table 3:** Docking and anticancer activity results of most active compounds and standard drug

Series	Comp. code	Molecular structure	IC_50_ (µM) HCT116	Docking score	Glide energy (kcal/mol)	Interacting residues	H-bonding with amino acids
I	**12**		9.53	− 8.907	− 57.165	Val35, Tyr32, Phe176, Asp173, Ala172, Leu95, Phe97, Asp98, Tyr 99, Ala100, Asp103, Ala155, Leu158, Glu66, Leu70, Lys 52, Ala50, Ile79, Val27, Gly28, Arg356	Asp173, Lys52, Ala100
II	**16**		11.97	− 7.69	− 57.228	Phe97, Asp98, Tyr99, Ala100, Val27, Asp103, His106, Ala155, Asn156, Leu158, Ala172, Asp173, Met174, Phe176, Glu66, Leu70, Ile79, Val35, Arg356, Tyr32, Ala50, Lys52	Ala155, Lys52
III	**N9**		5.85	− 7.425	− 53.041	Val35, Tyr32, Arg29, Gly28, Val27, Arg356, His106, Asp103, Ala100, Tyr99, Phe97, Phe176, Asp173, Ala172, Ala50, Lys52, Leu158, Asn156, Glu66, Leu70, Ile79	Tyr32,Val27, Lys52
IV	**W20**		11.61	− 9.686	− 52.697	Lys52, Ala50, Ile79, Phe97, Asp98, Tyr99, Ala100, Arg356, Asp103, Trp105, His106, Leu158, Asn156, Ala155, Tyr32, Val35, Val27, Asp173, Ala172	Ala100, Ala155
V	**Z24**		0.46	− 7.275	− 45.298	Ala50, Phe97, Asp98, Tyr99, Ala100, Leu359, Glu357, Arg356, Lys355, Ile79, Leu158, Trp105, His106, Val27, Gly20, Arg29, Tyr32, Val35,	Tyr32, Val27
Std.	**5-Fu**		8.84	− 5.79	− 21.629	Ile79, Ala50, Phe97, Asp98, Tyr99, Ala100, Arg356, Leu158, Val35	Asp98, Ala100

**Fig. 10:**
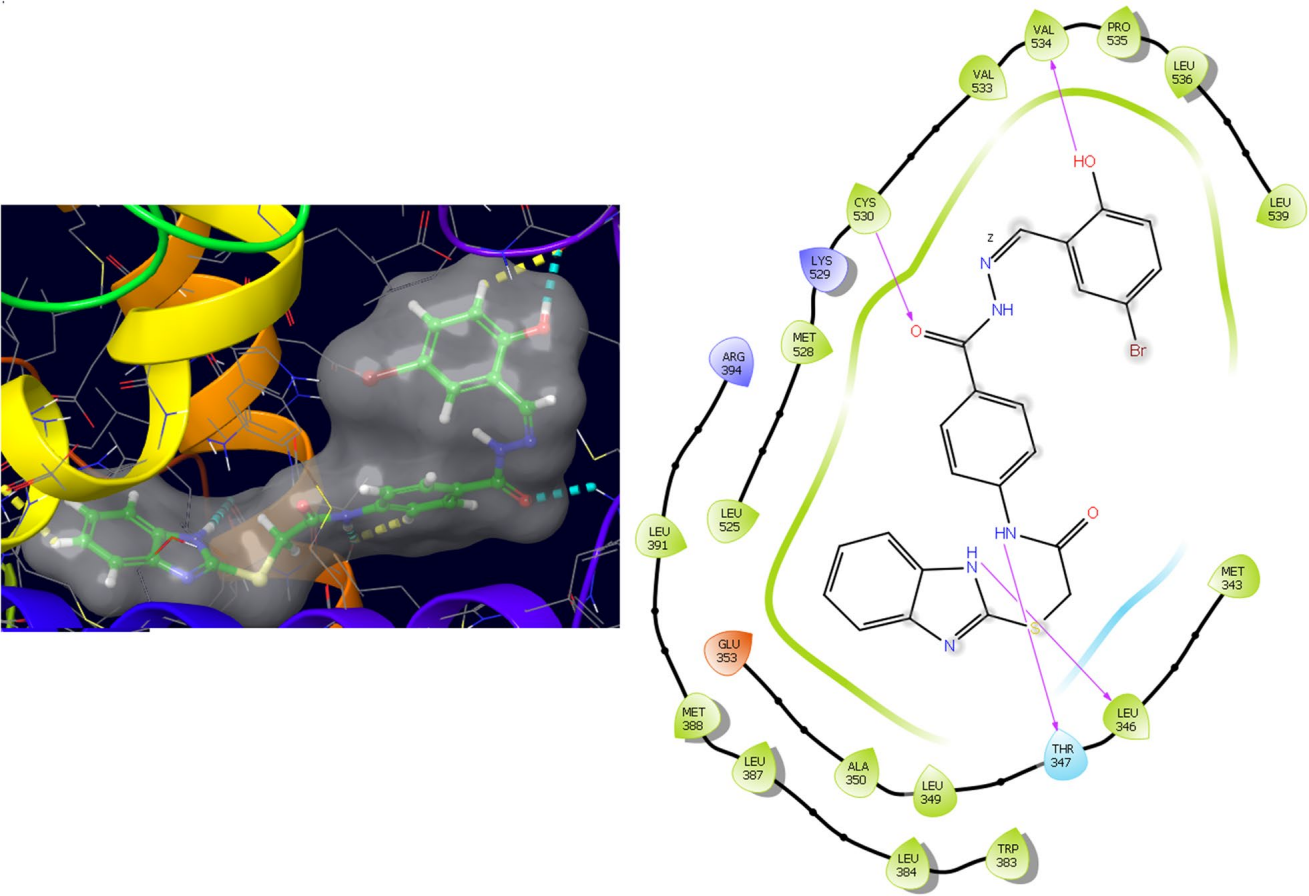
Binding surface and ligand interaction diagram of compound **12**

**Fig. 11:**
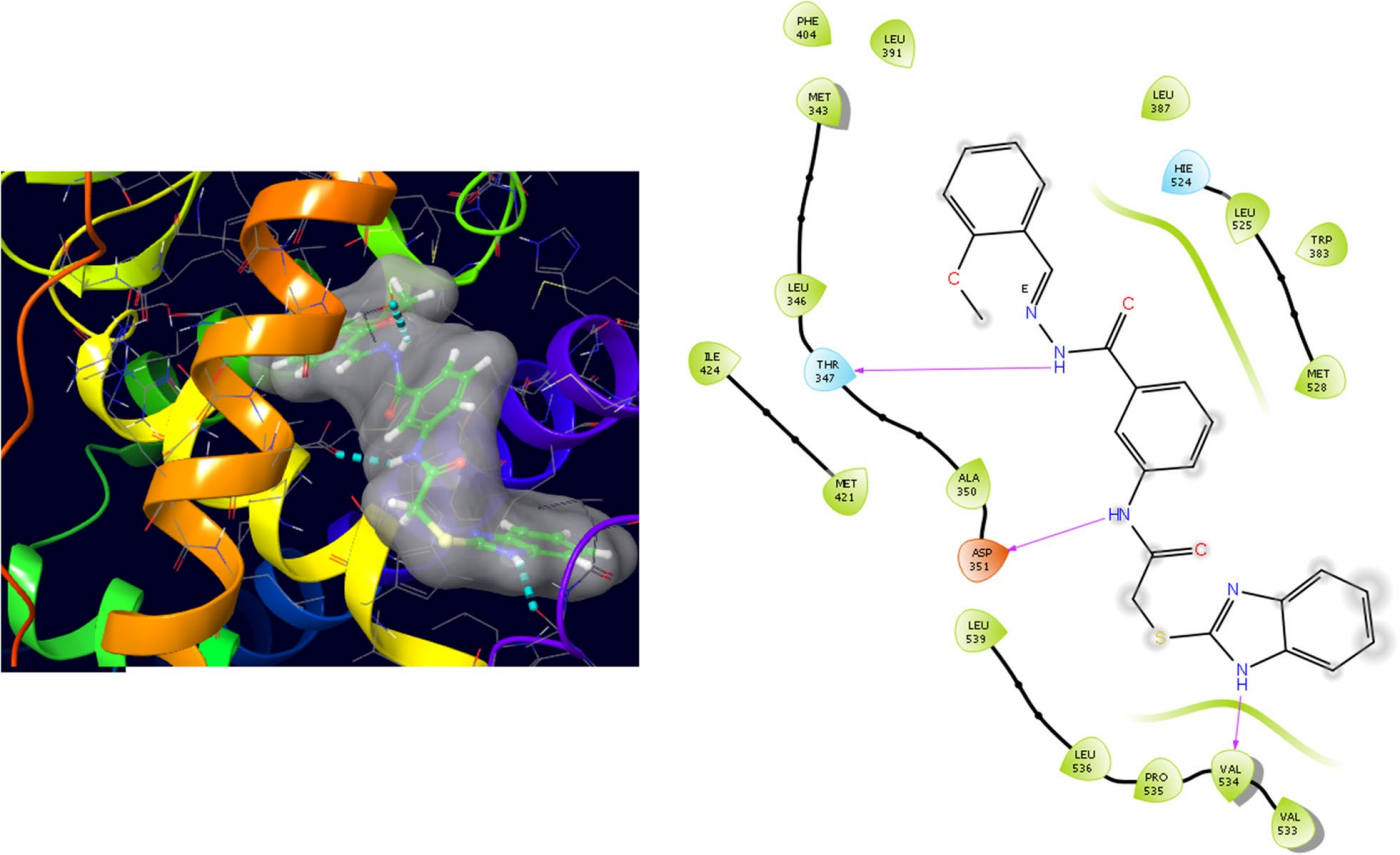
Binding surface and ligand interaction diagram of compound **16**

**Fig. 12:**
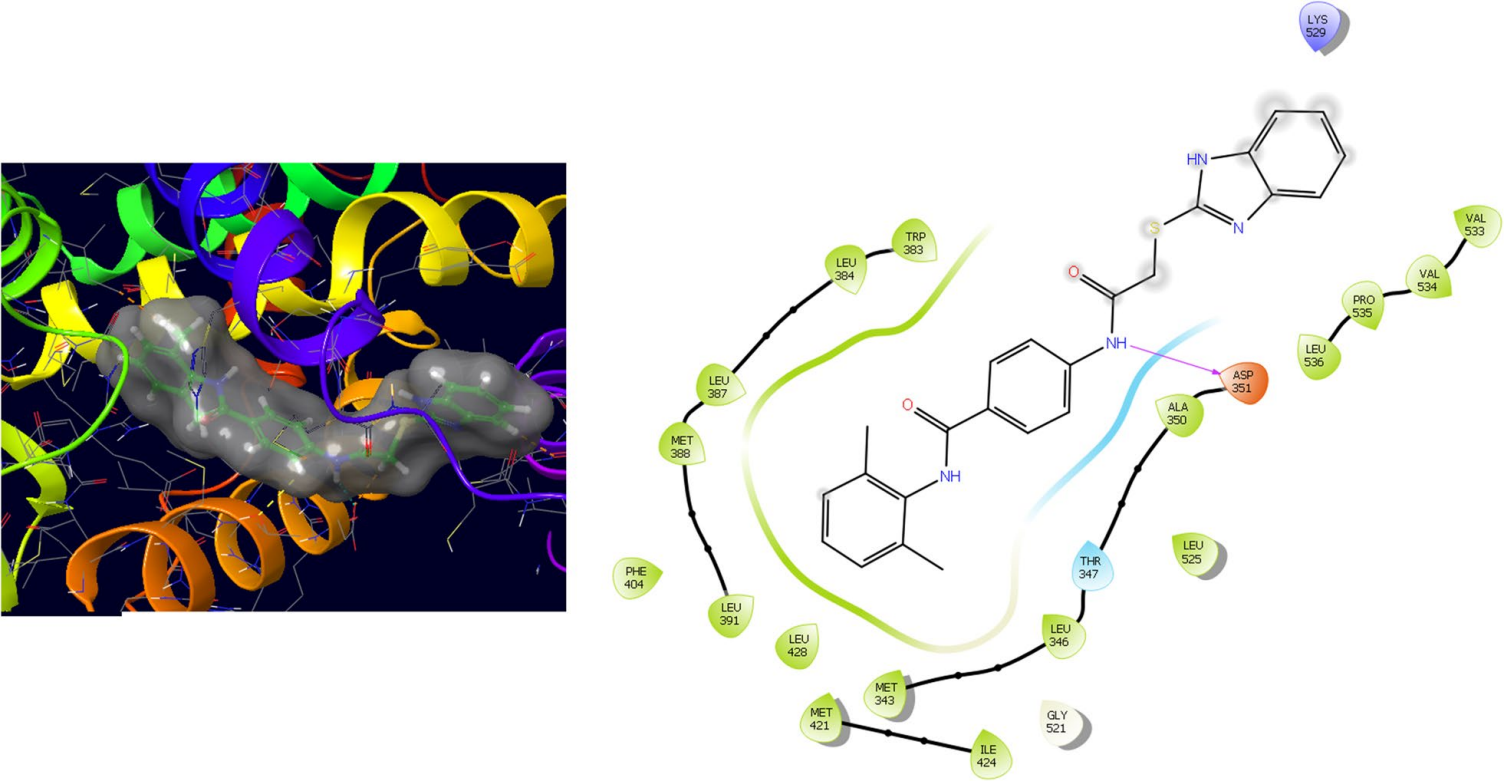
Binding surface and ligand interaction diagram of compound **N9**

**Fig. 13:**
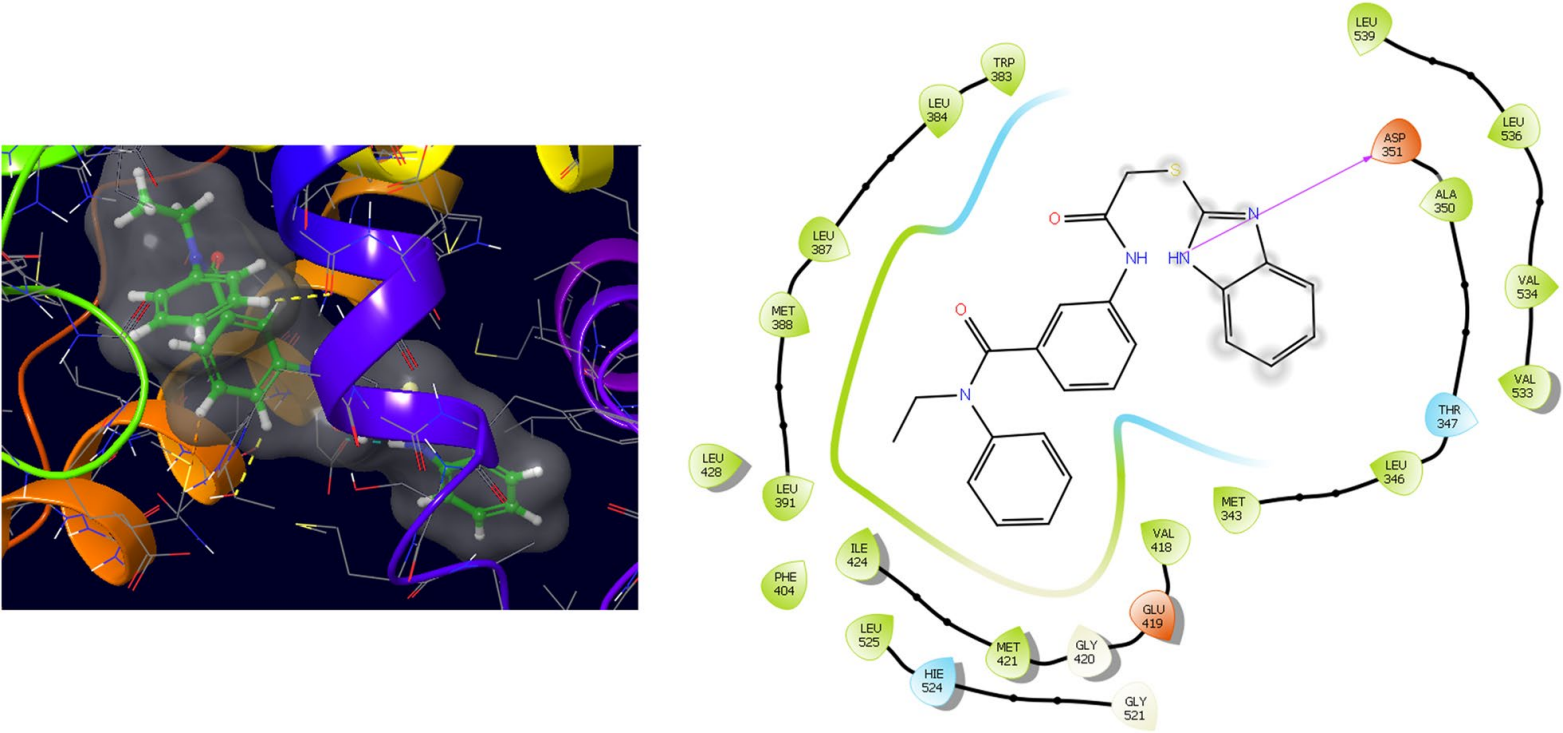
Binding surface and ligand interaction diagram of compound **W20**

**Fig. 14:**
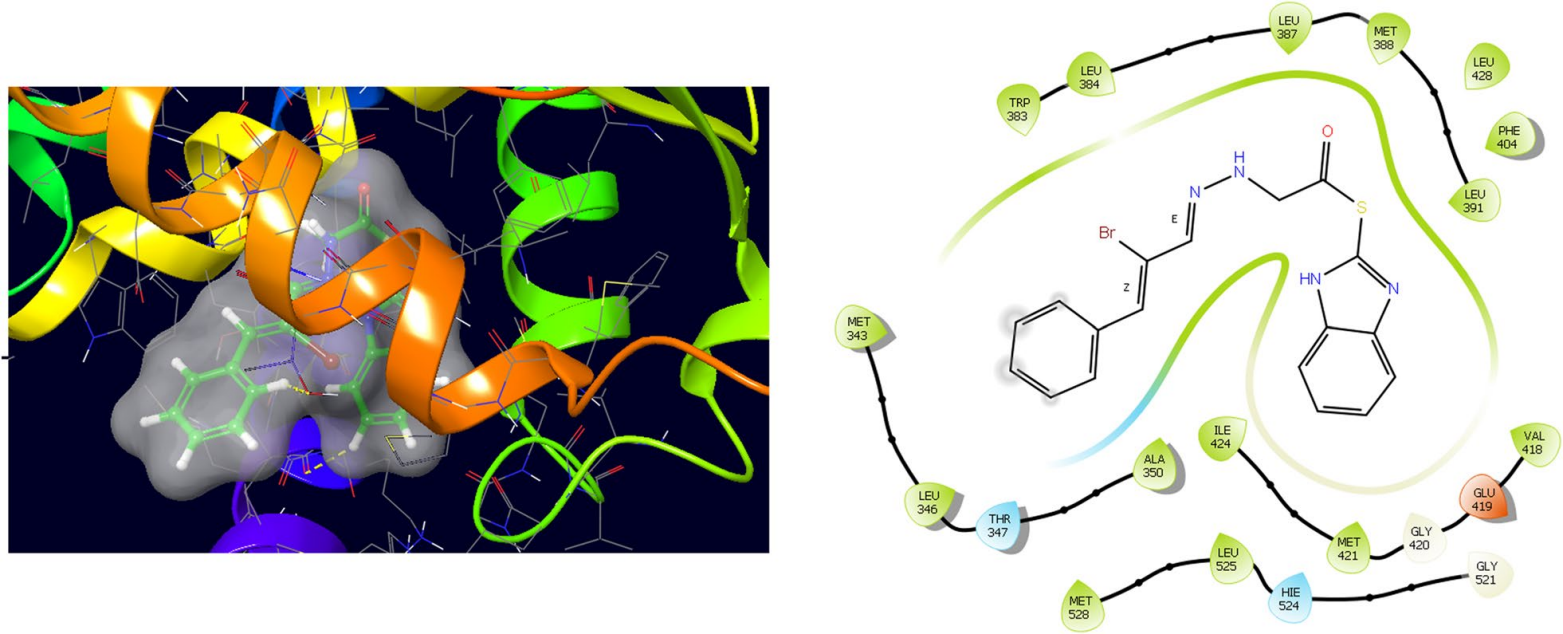
Binding surface and ligand interaction diagram of compound **Z24**

**Fig. 15:**
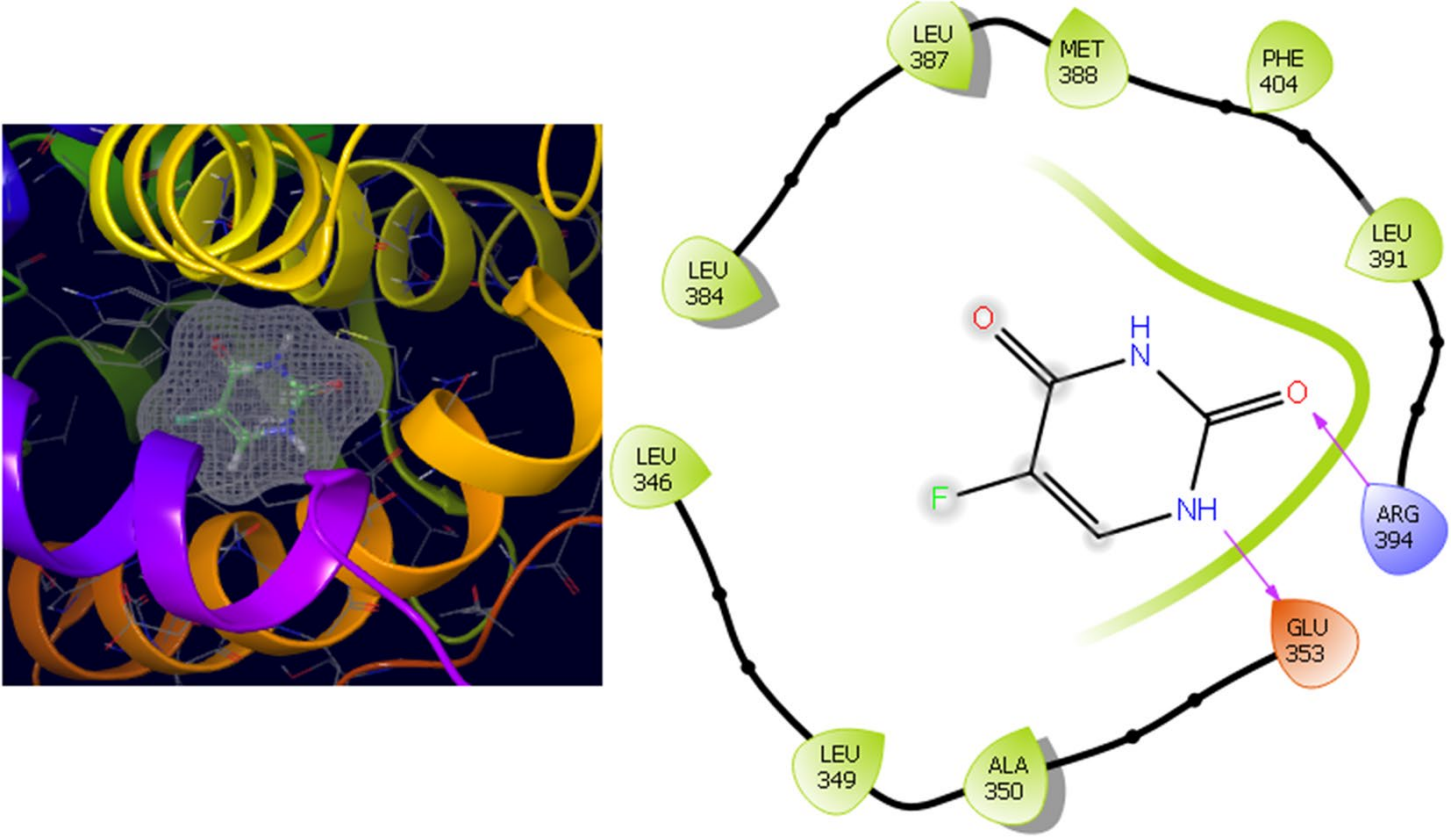
Binding surface and ligand interaction diagram of 5-fluorouracil

**Fig. 16:**
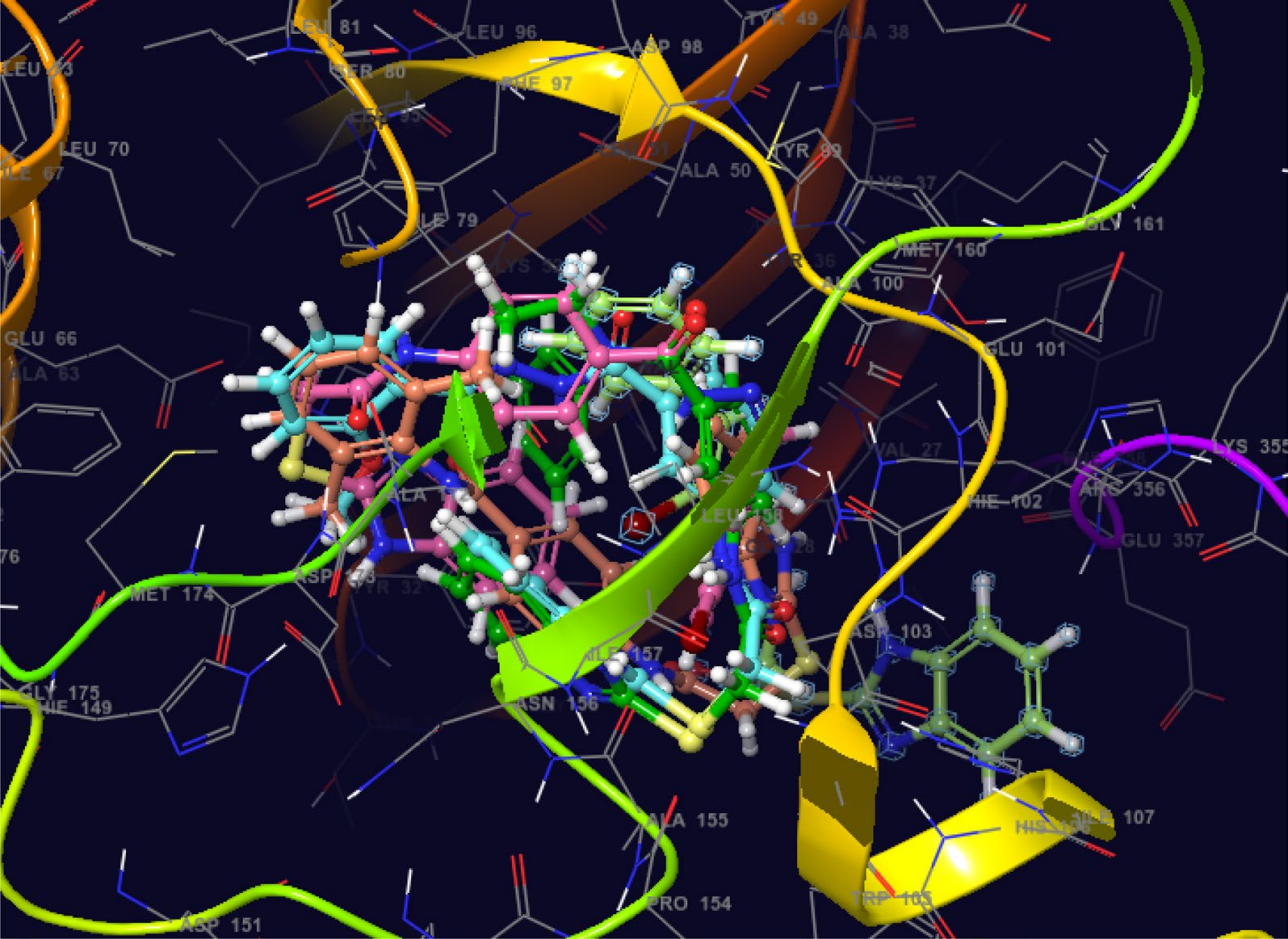
Binding mode of five most active compounds (**12**, **16**, **N9**, **W20** and **Z24**) into the CDK-8 active site

**Fig. 17:**
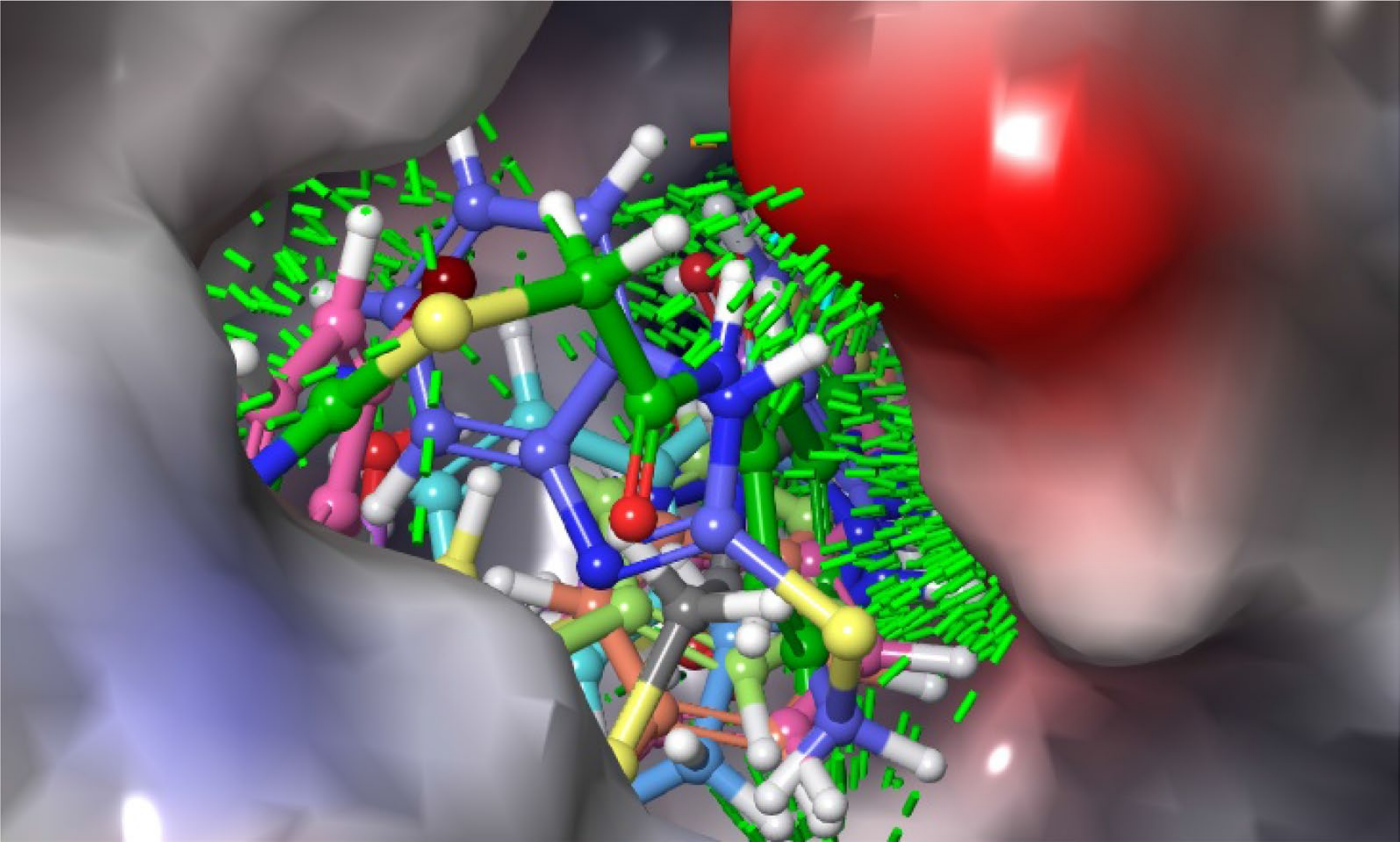
Binding mode of five most active compounds (**16**, **12**, **N9**, **W20** and **Z24**) into the 3ERT active site

**Fig. 18:**
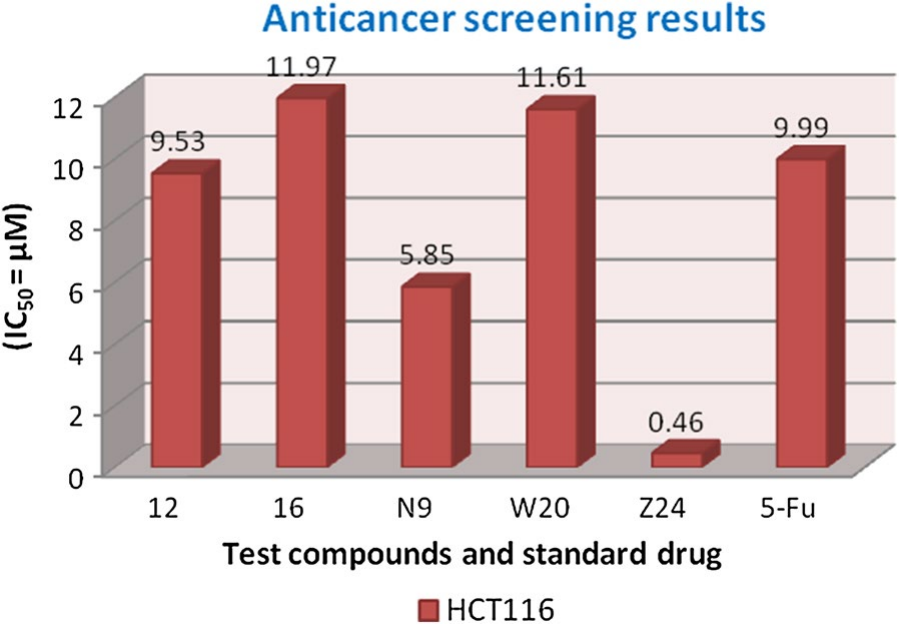
Anticancer screening results against HCT116 cancer cell line

**Fig. 19:**
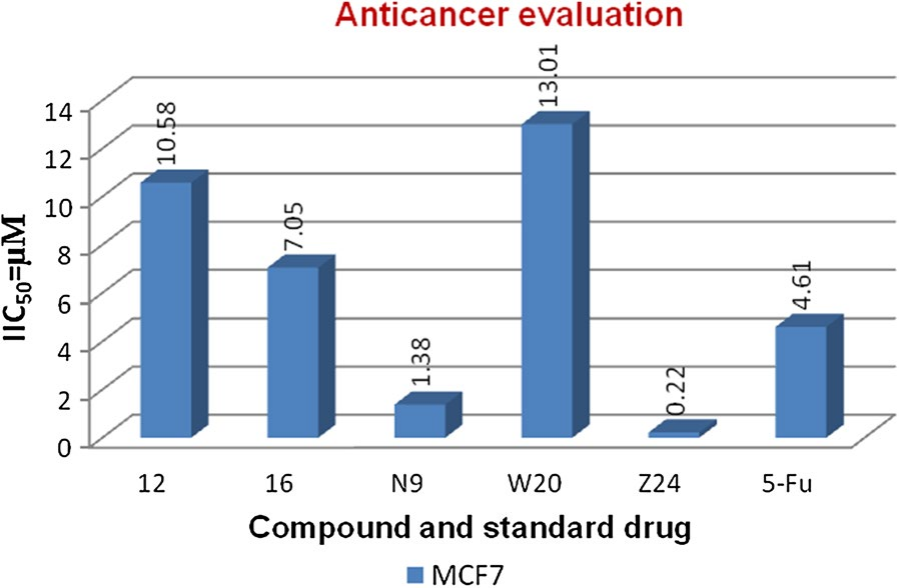
Anticancer screening results against MCF-7 cancer cell line

Further, the selected data set was docked with ER-alpha receptor of MCF7 (PDB id: 3ERT). Among the docked data set, compounds **12**, **16**, **N9**, **W20** and **Z24** also showed the good docked score and glide energy with crucial amino acids residues in the binding pocket of the receptor. If we look into the binding mode of compound **12** have docking score (− 8.825) and developed hydrogen bond with the crucial amino acids (Val534, Thr347, Leu346 and Cys530). The binding mode of compound **16** have docking score (− 8.986) and developed H-bond with amino acids Thr347, Asp351 and Val534. The binding mode of compound **N9** exhibited good docking score (− 6.748) and prepared hydrogen bond with crucial amino acid Asp351. Compound **W20** scored docking score (− 7.703) and prepared hydrogen bond with amino acids residues Asp351. Whereas, compound **Z24** displayed the good docking score (− 7.275) and prepared hydrogen bond within binding pocket. The standard drug (5-fluorouracil) have docking score (− 3.414) within the binding pocket. The docking results with interacting residues of the compounds and standard drug are shown in Table 4; binding surface (3D) and ligand interaction (2D) images are shown in Figs. 10, 11, 12, 13, 14, 15. Binding mode of five most active compounds is shown in Figs. 16 and 17. Based on the molecular docking analyses these compounds were evaluated for their in vitro anticancer activity against human breast adenocarcinoma cancer cell line (MCF7) by Sulforhodamine-B assay. The anticancer activity result of these compounds is shown in Tables 3 and 4, Figs. 18 and 19.

**Table 4:** Docking and anticancer activity results of most active compounds and standard drug

Series	Comp. code	Molecular structure	IC_50_ (µM) MCF7	Docking score	Glide energy (kcal/mol)	Interacting residues	H-bonding with amino acids
I	**12**		10.58	− 8.825	− 63.027	Leu539, Leu536, Pro535, Val534, Val533, Cys530, Lys529, Met528, Leu525, Glu353, Ala350, Leu349, Thr347, Leuy346, Met343, Trp383, Leu384, Leu387, Met388, Leu391, Arg394	Val534, Thr347, Leu346, Cys,530
II	**16**		7.05	− 8.986	− 54.764	Met343, Leu346, Thr347, Ala350, Asp351, leu539, Leu536, Pro535, Val534, Val533, Ile424, Met421, Phe404, Leu391, Leu387, Hie524, Leu525, Trp383, Met528,	Thr347, Asp351, Val534
III	**N9**		1.38	− 6.748	− 49.725	Trp383, Leu384, Leu387, Met388, Leu391, Phe404, Leu428, Met 343, Leu346, Thr347, Ala350, Asp351, Leu536, Pro535, Val534, Val533, Leu525, Gly521, Ile424, Met421	Asp351
IV	**W20**		13.01	− 7.703	− 58.783	Trp383, Leu384, Leu387, Met388, Leu428, Leu391, Phe404, Ile424, Met421, Gly420, Glu419, Val418, Leu525, Hie524, Gly521, Met343, Leu346, Thr347, Ala350, Asp351, Leu539, Leu536, Val534, Val533	Asp351
V	**Z24**		0.22	− 7.275	− 45.298	Trp383, Leu384, Leu387, Met388, Leu428, Pye404, Leu391, Met343, Leu346, Thr347, Ala350, Val418, Glu419, Gly420, Met421, Ile424, Gly521, Hie524, Leu525, Met528	–
Std.	**5-Fu**		4.61	− 3.414	− 24.58	Leu346, Leu349, Ala350, Glu353, Leu384, Leu387, Met388, Phe404, Leu391, Arg394	Glu353, Arg394

**Fig. 20:**
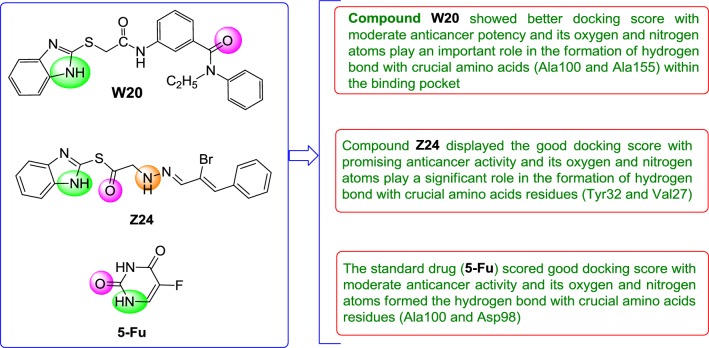
Structure activity relationship based on molecular docking study for HCT116 cell line

**Fig. 21:**
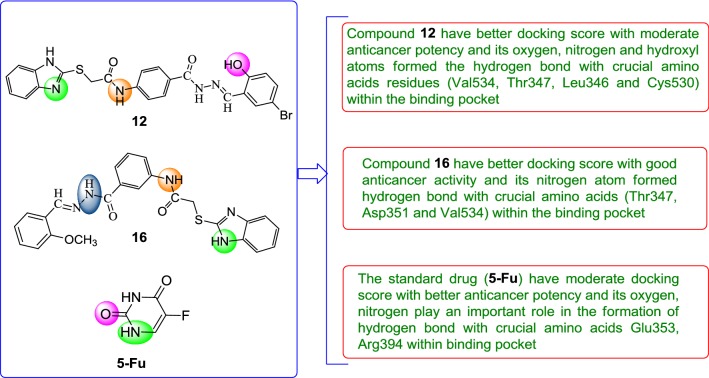
Structure activity relationship based on molecular docking study for MCF-7 cell line

Molecular docking results suggest that the selected compounds of heterocyclic benzimidazole can act as of great interest in successful chemotherapy. The selected protein data bank i.e. (PDB id: 5FGK and 3ERT) for human colorectal carcinoma and breast adenocarcinoma cancer cell lines may be the good target protein of benzimidazole molecules for their anticancer activity. Based on the docking analysis it is suggested that more structural modifications are required in molecules **12**, **16**, **N9**, **W20** and **Z2** to make them more potent toward cancer cell. The structure activity relationship study based on molecular doking is given in Figs. 20 and 21.

#### ADME results

Lipinski’s rule of five is a rule of thumb to evaluate drug likeness or determine if a chemical compound with a certain pharmacological or biological activity has chemical properties and physical properties that would make it a likely orally active drug in humans. The rule describes molecular properties important for a drug’s pharmacokinetics in the human body, including their absorption, distribution, metabolism, and excretion (ADME). The rule is important to keep in mind during drug discovery when a pharmacologically active lead structure is optimized step-wise to increase the activity and selectivity of the compound as well as to ensure drug-like physicochemical properties are maintained as described by Lipinski’s rule which states that (i) no more than 5 hydrogen bond donors, (ii) no more than 10 hydrogen bond acceptors, (iii) a molecular mass less than 500 daltons, (iv) an octanol–water partition coefficient log P not greater than 5 (https://en.wikipedia.org/wiki/Lipinski%27s_rule_of_five). Now these days, computational approaches are employed to determine the ADME of the drug molecules. ADME modeling has attracted the considerable attention of the pharmaceuticals researchers for the drug discovery as they are high-throughput in nature and cost effective [34]. ADME study of the selected compounds was performed using QikProp module of Maestro version *11.5*. Around eleven physically relevant and pharmacologically significant parameters of the most active compounds **16**, **N9** and **W20** were determined. The ADME results of the selected heterocyclic benzimidazole compounds **16**, **N9** and **W20** displayed the significant results within the close agreement with the Lipinski’s rule of five and Qikprop rule within the range i.e. Molecular weight of the molecule (mol. MW ≤ 500), Predicted octanol/water partition coefficient (QPlogPo/w = − 2.0 to − 6.5), (QPlogPw = 4.0 to − 45.0), Predicted water/gas partition coefficient (QPlogKp = − 8.0 to − 1.0), Predicted brain/blood partition coefficient (QPlogBB = − 3.0 to − 1.2), donor HB (0.0 to − 6.0), accept HB (2.0 to − 20.0), human oral absorption (1, 2 or 3), percent human oral absorption (0 to 100), Predicted water/gas partition coefficient thus making these compounds as suitable drug candidate. The ADME results are shown in the in Table 5.

**Table 5:** ADME parameters of the most active compounds

S. no.	Comp.	Molecular structure	ADME parameters										
			Mol MW	Rule of five	QPlogPo/w	Human oral absorption	Volume	Percent human oral absorption	QPlogP_w_	QPlogK_p_	QPlogBB	Donor HB	Accept HB
1	**16**		459.5	0	4.681	1	1454.82	100.0	15.86	− 1.699	− 1.812	3.0	7.25
2	**N9**		430.5	0	4.748	1	1379.64	100.0	15.049	− 1.438	− 1.054	3.0	6.5
3	**W20**		430.5	0	4.903	1	1386.55	100.0	14.002	− 1.028	− 0.975	2.0	7.0

## Conclusion

In the present work the molecular docking study of the data set of heterocyclic benzimidazole molecules was performed by Maestro version *11.5.* In this study we have used CDK8 for human colorectal carcinoma cancer and ER-alpha for breast adenocarcinoma cancer cell lines and identified best anticancer target. Molecular docking results displayed that compounds (**12**, **16**, **N9**, **W20** and **Z24**) showed the better docked score with moderate to better antiproliferative potency towards cancer cell line within the binding pocket of receptor and comparable to the standard drug. The compounds **16**, **N9** and **W20** also showed good ADME properties within the close agreement of the Lipinski’s rule of five and Qikprop rule within the range and thus making these compounds as suitable drug molecules.

## Data Availability

We have presented all our main data in the form of tables and figures.

## Abbreviations

ADME: absorption, distribution, metabolism and excretion
CDK8: cyclin dependent kinase 8
ER-alph: estrogen receptor alpha
HCT116: human colorectal carcinoma 116
MCF-7: Michigan Cancer Foundation-7
μM: micro mole
PDB: protein data bank
XP: extra precision
5-Fu: 5-fluorouracil
H-bond: hydrogen-bond
2D: 2 dimensional
3D: 3 dimensional
RMSD: root-mean-square deviation
